# Strategies for Diagnosis and Treatment of Suspected Leptospirosis: A Cost-Benefit Analysis

**DOI:** 10.1371/journal.pntd.0000610

**Published:** 2010-02-23

**Authors:** Yupin Suputtamongkol, Wirichada Pongtavornpinyo, Yoel Lubell, Chuanpit Suttinont, Siriwan Hoontrakul, Kriangsak Phimda, Kitti Losuwanaluk, Duangjai Suwancharoen, Saowaluk Silpasakorn, Wirongrong Chierakul, Nick Day

**Affiliations:** 1 Faculty of Medicine Siriraj Hospital, Mahidol University, Bangkok, Thailand; 2 Mahidol-Oxford Tropical Medicine Research Unit, Faculty of Tropical Medicine, Mahidol University, Bangkok, Thailand; 3 Maharat Nakhon Ratchasima Hospital, Nakhon Ratchasima Province, Thailand; 4 Chumphon Hospital, Chumphon Province, Thailand; 5 Udon Thani Hospital, Udon Thani Province, Thailand; 6 Banmai Chaiyapod Hospital, Burirum Province, Thailand; 7 The National Institute of Animal Health, Ministry of Agriculture and Cooperative, Bangkok, Thailand; Institut Pasteur, France

## Abstract

**Background:**

Symptoms and signs of leptospirosis are non-specific. Several diagnostic tests for leptospirosis are available and in some instances are being used prior to treatment of leptospirosis-suspected patients. There is therefore a need to evaluate the cost-effectiveness of the different treatment strategies in order to avoid misuse of scarce resources and ensure best possible health outcomes for patients.

**Methods:**

The study population was adult patients, presented with uncomplicated acute febrile illness, without an obvious focus of infection or malaria or typical dengue infection. We compared the cost and effectiveness of 5 management strategies: 1) no patients tested or given antibiotic treatment; 2) all patients given empirical doxycycline treatment; patients given doxycycline when a patient is tested positive for leptospirosis using: 3) lateral flow; 4) MCAT; 5) latex test. The framework used is a cost-benefit analysis, accounting for all direct medical costs in diagnosing and treating patients suspected of leptospirosis. Outcomes are measured in length of fever after treatment which is then converted to productivity losses to capture the full economic costs.

**Findings:**

Empirical doxycycline treatment was the most efficient strategy, being both the least costly alternative and the one that resulted in the shortest duration of fever. The limited sensitivity of all three diagnostic tests implied that their use to guide treatment was not cost-effective. The most influential parameter driving these results was the cost of treating patients with complications for patients who did not receive adequate treatment as a result of incorrect diagnosis or a strategy of *no-antibiotic-treatment*.

**Conclusions:**

Clinicians should continue treating suspected cases of leptospirosis on an empirical basis. This conclusion holds true as long as policy makers are not prioritizing the reduction of use of antibiotics, in which case the use of the latex test would be the most efficient strategy.

## Author Summary

Symptoms and signs of leptospirosis are non-specific. A number of diagnostic tests for leptospirosis are available. We compared the cost-benefit of 5 management strategies: 1) no patients tested or given antibiotic treatment; 2) all patients given empirical doxycycline treatment; patients given doxycycline when a patient is tested positive for leptospirosis using: 3) lateral flow; 4) MCAT; 5) latex test. Outcomes were measured in duration of fever which is then converted to productivity losses to capture the full economic costs. Empirical doxycycline treatment was found to be the most efficient strategy, being both the least costly alternative and the one that resulted in the lowest average duration of fever. The significantly higher relative cost of using a diagnostic test as compared with presumptive treatment, and the limited sensitivity of all the diagnostic tests implied that only the latex test could be considered cost-effective when compared with the *no-antibiotic-treatment* option, and that all three tests were still inferior to empirical treatment.

## Introduction

Leptospirosis is a zoonosis of worldwide distribution, caused by infection with pathogenic spirochetes of the genus *Leptospira*. Human leptospirosis is an important health problem in Asia [1]–[3] and Latin America [4],[5]. The source of infection in humans is either direct or indirect contact with the urine of an infected animal, whether livestock, domestic pets, rodents or wild animals. Veterinarians, abattoir workers and other occupations which require contact with animals are at risk of infection. Indirect contact can also cause infection in occupations such as rice field workers, sewer workers, and soldiers. Peak incidence occurs during the rainy season in tropical regions. Clinical manifestations of leptospirosis are non-specific, varying from subclinical infection, through self-limited anicteric febrile illness with or without meningitis, to severe and potentially lethal multisystem illness with jaundice and renal failure [6],[7].

Recent findings show that leptospirosis is a common cause of undifferentiated febrile illness in developing countries [3],[8],[9]. Early diagnosis of leptospirosis is essential since antibiotic therapy provides greatest benefit when initiated early in the course of illness [6],[7]. Diagnosis at an early phase, however, is hampered by the non-specific presentation of leptospirosis. A number of diagnostic tests for leptospirosis are available, all of these test are aimed to detect specific antibody against pathogenic *Leptospira*. These tests have so far shown low levels of accuracy, questioning their usefulness in the selection of appropriate antimicrobial treatment [10]. Nonetheless, these tests are often used in routine practice in many clinical settings. The objective of this study is therefore to determine whether it is cost-effective to perform these screening tests for leptospirosis in patients with acute febrile illness suspected of mild leptospirosis, and if so, which diagnostic assay is most cost-effective, in the outpatient setting.

## Methods

The economic framework used in this study is a cost-benefit analysis, where the direct diagnosis and treatment costs are compared with productivity gains under the different diagnosis and treatment strategies. The perspective of the analysis is therefore a societal one, capturing both provider costs and patient productivity gains.

While much of the data were collected in a concurrent clinical study [11], the study population in this analysis was a hypothetical cohort of adult patients (>14 years) who present with acute fever (<15 days) suspected of leptospirosis i.e., no obvious focus of infection, without severe complications or impaired consciousness, and are suitable for oral antimicrobial therapy. Malaria and obvious cases of dengue infection, and pregnant women are excluded.

We compared the cost and outcomes of five management strategies. Under the first strategy patients were not given antibiotic treatment; this provides the baseline with which other strategies are compared in terms of their incremental costs and outcomes. Under the second strategy all patients were given empirical treatment consisting of a 7-day course of doxycycline, 100 mg bid treatment. Strategies 3 to 5 consisted of provision of a 7-day course of doxycycline to patients with a positive test result using either a lateral flow test, a microcapsule agglutination test (MCAT), or a latex test. Patients with a negative test result were assumed to receive no antibiotic treatment. Patients given doxycycline could experience antibiotic side effects such as rash or gastrointestinal distress. Patients with leptospirosis or rickettsial infection (scrub typhus or murine typhus) who did not receive an antibiotic could develop a serious disease complication, such as renal failure or aseptic meningitis.

The primary measure of effectiveness was the duration of fever following the hospital visit, which in turn determined the productivity losses for days off work. The reduction in productivity losses following either empirical treatment or the use of the diagnostic tests was compared to the productivity losses of the *no- antibiotic -treatment* strategy; these provided the productivity gains associated with each strategy. For direct medical costs we used charges, rather than costs. These were applied to the cost of initial diagnosis and treatment and the subsequent treatment of any adverse reactions to the drugs and further complications to patients that did not receive appropriate initial treatment.

The costs and productivity gains for each strategy were then compared. Where a strategy is both less costly and more effective than the *no-antibiotic-treatment* option it clearly dominates. Where one strategy incurs higher costs and higher benefits than the baseline (or any other comparator) its efficiency is calculated using a benefit-cost ratio (BCR), a product of the difference in costs divided by the difference in outcomes. Where the BCR is above 1 the intervention can be considered cost-effective.

### Decision Model

**Figure 1:**
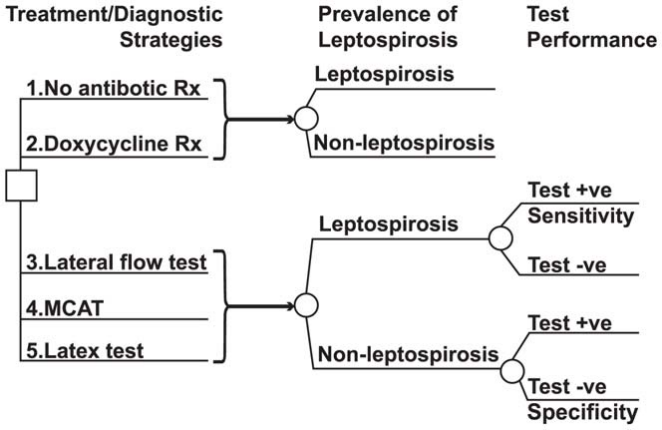
The decision model showing each of the treatments and diagnostic options (first five branches). The square at the far left of the diagram represents a decision node, with each branch representing the clinical management choices. The brackets indicate that patients who received each of the strategies proceeded to the subtree to the right. The circles at the start of each subsequent branching indicate chance nodes representing the uncertainty surrounding possible subsequent outcomes indicated in the branches to the right. This diagram shows the portion of the decision tree modeling the management strategies, the prevalence of leptospirosis, and diagnostic test performance (sensitivity and specificity).

We developed a decision tree with Markov nodes [12] to compare the costs and outcomes of a hypothetical cohort of 10,000 patients with suspected leptospirosis under each of the strategies (Figure 1). All patients began the simulation with acute fever and symptoms suggestive of leptospirosis. The model simulated the natural history of suspected leptospirosis progression over a 7-day period, during which the patients make various possible transitions; if they are sick, they may remain sick, become well, become sick with an antibiotic side effect, or develop a serious disease complication. A 7-day limit was applied because most patients became afebrile within this period, as shown in our concurrent clinical study [11]; the remaining proportion of febrile patients at the end of the 7 day period was also calculated. The model estimated the number of fever days associated with each strategy by summing the daily proportions of patients without fever. Given the short time horizon of 7 days, costs and outcomes were not discounted.

#### Assumptions of the model

We made the following assumptions in structuring the model:

1. Once resolved, symptoms did not recur during the study period;
2. The daily risk of antibiotic side effects remained constant during the 7-day course. Side effect occurred only once and required symptomatic treatment, but did not alter the cure rate;
3. Patients with leptospirosis that received treatment could not develop a severe complication.

### Data Summary

Limited information was available for many of the parameters in the analysis. Where possible, we used the results of our concurrent clinical study of adult patients with acute undifferentiated febrile illness suspected of leptospiorsis [11. This study was conducted between July 2003 and January 2005 at 5 hospitals in Thailand. The results of this study showed that leptospirosis and rickettsial infection (mainly scrub typhus and murine typhus) had similar clinical manifestations, and accounted for approximately 50% of the cause of acute undifferentiated fever. This was supplemented where necessary with further data from the literature and expert opinion. Tables 1 through [Table pntd-0000610-t002] [Table pntd-0000610-t003] 4 list the values used for the variables in the model, the range of values tested in the sensitivity analyses, and the data sources.

**Table 1:** Values and sources of inputs used in the decision model.

Input	Base Case Value, %
Leptospirosis prevalence [11]	26
Antibiotic side effect (rate) [11]	24.3
Complication due to untreated leptospirosis [16]–[19]	10
Complication due to untreated rickettsial infection	10
Complication due to other undifferentiated untreated illnesses [11]	8.4

**Table 2 pntd-0000610-t002:** Test performance using acute sera [10],[14].

Test	Base case	Sensitivity Analysis Range	
		(Lower bound)	(Upper bound)
**Lateral flow test**			
Sensitivity	0.34	-	0.70
Specificity	0.88	0.83	0.95
**MCAT**			
Sensitivity	0.36	0.26	0.81
Specificity	0.91	0.73	0.95
**Latex test**			
Sensitivity	0.51	0.35	0.74
Specificity	0.73	0.60	0.95

**Table 3 pntd-0000610-t003:** Outcome of treatment.

		Data Distribution	Base Case Value, day (SD)	Sensitivity Analysis Value
In leptospirosis	Duration of fever with doxycycline treatment[11],[19]	Normal	2.04 (1.04)	0.33–4.92
	Duration of fever without doxycycline treatment [19]	Normal	5.4 (0.3)	-
In non-leptospirosis: Rickettsial infection	Duration of fever with doxycycline treatment [11]	Lognormal	1.62(0.67)	0.5–4.5
In non-leptospirosis: Others	Duration of fever with doxycycline treatment [11]	Lognormal	2.67 (2.46)	0.33–15.8
	Duration of fever without doxycycline treatment [11],[18]	Normal	5.3 (1.3)	-

**Table 4:** Costs (USD).

	Base Case Value, USD
Doxycycline prescription	2^a^
Lateral flow test	5.7^b^
MCAT	5.7^b^
Latex test	5.7^b^
Doxycycline side effect, per day	
- no work loss	2.9^c^
- work loss	5.25
Disease outcome (at end of 7-day course)	
- Cure	0
- Sick (daily cost of work loss from leptospirosis)	5.25
- Serious complication	341^d^

a Cost of doxycycline (100 mg) 14 capsules in Thailand

b Cost as of 2004 of lateral flow, MCAT, latex test.

c Treatment of side effect symptoms such as antiemetic drug or antihistamine for rash.

With work loss, 1 day at minimum earning (5.25 USD) assumed.

There is no additional cost to patients not receiving antibiotics or to being cured.

d Estimate of hospital costs, including intravenous antibiotics, other treatment.

### Diagnostic Tests

*Lateral flow* (Lepto Tek, BioMerieux, The Netherlands) is a one step colloidal gold immunoassay. It is based on the binding of specific IgM antibodies to the broadly reactive heat- extracted antigen prepared from the non-pathogenic Patoc 1 strain [13]. This test was first tested in south Andaman, India in 2003 [13].

*Microcapsule agglutination test* (MCAT, Japan Lyophilization Lab. Tokyo, Japan) is a passive agglutination assay, using microcapsule particles of a stable synthetic polymer to which surface cell components of mixture of 6 sonicated *Leptospira* spp. (serogroup Australis, Autumnalis, Hebdomadis, Canicola, Icterohaemorrhagia, and Pyrogenes) are adsorbed [14]. This test was developed and first tested in Japan and had been used in Thailand since 1997 [15].

*Latex agglutination test* (National Institute of Health, Ministry of Public Health of Thailand) is a latex agglutination test to detect Leptospira-specific antibodies. This test was developed by National Institute of Health, Ministry of Public Health of Thailand and has been used in Thailand since 2001 [16].

These three tests are widely used for the diagnosis of leptospirosis in Thailand. Field evaluations in endemic areas and in a clinical study in Thailand indicated that their performance was characterized by low sensitivities during acute-phase illness [10],[11],[13]. The data are shown in table 2. The costs of these tests were similar.

### Prevalence

Prevalence estimates of leptospirosis in different settings varied from 7–36.9% [3],[8],[9],[11]. The prevalence of leptospirosis in our latest concurrent clinical study was 26% [11], which we used as a baseline in this study.

### Complications

We considered any major organ dysfunction such as acute renal failure, hypotension, acute respiratory failure [17] associated with leptospirosis to be a proxy for severe disease. Although mild leptospirosis could be self-limiting, complications of untreated leptospirosis occurred between 10–28% [11], [18]–[20]. We used a baseline estimated of 10%.

### Treatment of Suspected Leptospirosis Patients and Their Outcomes

We used data from our concurrent clinical study [11] and findings from the literature on treatment options and their outcomes [21]. Among patients who presented with acute undifferentiated fever suspected of being leptospirosis, duration of fever and rate of complications in patients without leptospirosis or scrub typhus that did not receive doxycycline therapy were unknown, and were estimated to be similar to patients who had leptospirosis but did not receive doxycycline.

## Results

### Clinical Effectiveness Profile

Not providing antibiotic treatment to any patient yielded the longest average duration of fever of 5.35 days (95%CI 5.32–5.39), and the worst cure rate, but avoided all antibiotic side effects. Empirical antibiotic treatment yielded the shortest duration of fever, averaging 2.24 days (95%CI 2.23–2.25), the highest cure rate at the end of the first week, and avoided any complications; it also, however, led to the highest rate of antibiotic side effects (Table 5). The application of lateral flow, MCAT or latex tests yielded average durations of fever of 4.66 (95%CI 4.70–4.89), 4.83 (95%CI 4.80–4.87) and 4.30 (95%CI 4.25–4.34), days respectively. Between 5–9% of patients developed complications, dependent on the exact test sensitivity and specificity.

**Table 5:** Costs, clinical outcomes and antibiotic prescription at various prevalence rates and for patients receiving no treatment, empirical treatment, diagnosis using the latex test and diagnosis with a test with 95% sensitivity and specificity.

	Average cost/patient (USD)	Health outcomes			Doxycycline Prescriptions	
		Duration of fever (Patients with fever at D7, %)	Patients with side effect, %	Patients with complications, %	Patients without leptospirosis given doxycycline, %	Patients with leptospirosis not given doxycycline, %
**10% prevalence**						
No antibiotic Rx	13.3	5.35 (5.8)	0	9.3	0	100
Empirical Rx	2.7	2.29 (1.7)	24	0	100	0
Latex test	15.7	4.44 (4.6)	7	6.47	27	49
High accuracy test	17.22	4.88 (5.54)	3	7.7	5	5
**26% prevalence**						
No antibiotic Rx	13.3	5.35 (4.7)	0	9.3	0	100
Empirical Rx	2.7	2.24 (1.3)	24	0	100	0
Latex test	15.4	4.30 (3.8)	8	6.2	27	49
High accuracy test	15.75	4.38 (4.53)	7	6.5	5	5
**35% prevalence**						
No antibiotic Rx	13.3	5.35 (4.26)	0	9.3	0	100
Empirical Rx	2.7	2.22 (1.22)	24	0	100	0
Latex test	15.25	4.23 (3.42)	9	6.0	27	49
High accuracy test	14.93	4.14 (4.07)	9	5.8	5	5

### Costs

The baseline strategy of no-antibiotic- treatment incurred an average cost of 13.3 USD per patient. With no initial diagnosis or treatment costs, this expenditure is due entirely to patients that develop complications, not having received initial treatment. Empirical treatment of all patients incurred far lower expenditure than the other strategies, with an average cost per patient of 2.7 USD. This is comprised mostly (74%) of the initial treatment cost, with the remaining expenditure due to the treatment of side-effects. The diagnostic tests all incurred similar costs, between15.3 and 17.2 USD. These costs were largely driven by the treatment of severe complication (approximately 60%), with the tests themselves being the second largest component (approximately 35%).

When comparing the costs and productivity gains of the different strategies, empirical treatment clearly dominates the rest, being both the cheapest and most effective option. When compared to the no-antibiotic-treatment option, use of all three diagnostic tests provides some productivity gains, but also incurs higher costs. Accounting for the differences in costs and productivity, only the latex test had a BCR above one (2.68), while the BCRs for the lateral flow and MCAT tests were 0.71 and 0.75, respectively (Table 6).

**Table 6:** Benefit-cost ratios for the different strategies.

Strategy	Direct costs (USD)	Productivity loss (USD)	Benefit-cost ratio
No –antibiotic- treatment	13.26	28.4	(Baseline)
Empirical treatment	2.70	11.8	−1.57*
Latex test	15.41	22.6	2.68
Lateral flow test	17.30	25.5	0.71
MCAT	17.23	25.4	0.75

*: The negative value is a result of the empirical treatment strategy being both less expensive and more effective than the *no –antibiotic*- treatment baseline

### Sensitivity Analyses

We performed sensitivity analyses to examine the effect of varying parameter values used in the analysis as specified in tables 2 and 3 and using a leptospirosis prevalence of 10% and 35%; this did not significantly alter results therefore these are not presented in detail. With lower or higher prevalence, empirical treatment remained the least costly and most effective strategy. Increased test sensitivity and specificity to 95% had little impact on overall costs and outcomes (Table 5). Increase test accuracy in the high leptospirosis prevalence area has much more impact when compared with the lower leptospirosis prevalence area, in terms of cost per patient (14.93 vs. 17.22 USD), duration of fever (4.14 vs. 4.88 days), percentage of patients with fever at day 7 (4.14% vs. 4.88%) and percentage of patients with complications (5.8% vs. 7.7%). Varying the costs of the tests and of doxycycline within a reasonable range did not alter the results in favor of either of the tests; in fact doxycycline would have to cost over 29 USD, well beyond its current price, for the latex test to become a more efficient strategy.

## Discussion

Leptospirosis has become an important public health problem worldwide [22]. Much emphasis has been placed on the development of improved serologic tests that use whole cell Leptospira antigen preparations. Commercial whole –based assays are available in rapid formats amenable for ‘point- of- care’ use. Field evaluations indicate that these assays are characterized by low sensitivities (39–72%) during acute – phase illness [10],[22]. Lateral flow, MCAT, and latex tests are widely used assays for the diagnosis of leptospiorsis in Thailand. Because of their relatively high costs and low sensitivities, use of these tests for the initial management of acute leptospirosis was inferior to empirical treatment, and only the latex test was cost-effective when compared to the no-antibiotic- treatment option.

Empirical treatment with doxycycline was found to be the most cost-effective strategy, being both cheap and effective in treating uncomplicated leptospirosis and other causes of febrile illness. Results from our concurrent clinical study showed that acute undifferentiated fever, i.e. acute fever without an obvious focus of infection, is the most common clinical presentation of both leptospirosis and scrub typhus [11]. Antibiotic treatment with either doxycycline or azithromycin shortened the duration of fever in both these and other illnesses. The use of a diagnostic test therefore implies that where a test provides a negative result, true or false, the patient would be denied a potentially effective treatment.

Treatment failure occurred in 2% of intended-to- treat patients. The limitation of doxycycline empirical therapy was nausea/vomiting which occurred in about 24.3% of doxycycline treated patients and severe adverse events (rash and severe vomit) developed in 2 out of 145 treated patients [11].

Other epidemiological studies showed that “systemic infection” such as dengue infection, rickettsial infection, brucellosis, Q fever, CMV or EBV infection was common causes of acute fever syndrome, and in many cases fever disappeared without specific diagnosis being established [23]. Doxycycline was the most common empirical antimicrobial therapy for this syndrome and all episodes resolved without further complications. Shorter duration of fever among those patients who received treatment was also observed [23].

A primary limitation of this analysis is that it did not account for the potential harm to society that results in the overuse of doxycycline, which could eventually lead to increased bacterial resistance, reduced future effectiveness, and increased drug costs. Leptospiral resistance to doxycycline has not been described. More studies to clarify the link between individual antibiotic use and emerging community resistance are needed. If policy makers wish to prioritize the reduction in antibiotic use with the use of a diagnostic test, this study shows that the use of the latex test can be considered cost-effective.

The data used in this analysis concerning doxycycline treatment was determined from our prospective clinical study [11]. The data used for the *no-antibiotic*-*treatment* group on the other hand was limited by its reliance on published data and expert opinion. The sensitivity analysis however showed that variation in these parameters, within reason, did not have a significant impact on results. Lastly, the costs for direct medical expenses was based on charges, rather than actual economic costs; based on familiarity with the Thai healthcare system we assume that actual costs are higher, therefore the cost-effectiveness of the intervention is likely to be slightly higher than that found in the analysis.

In summary leptospirosis is a common cause of acute undifferentiated fever in rural areas where limited resources are available. Results of this study and other clinical studies [11],[23] show that empirical treatment with doxycycline would be the most cost-effective option for these patients, as this strategy was also beneficial for patients with other diseases which clinically mimic leptospirosis such as scrub typhus. It should be noted that this strategy applies only to adult patients with acute fever suspected of mild leptospirosis. Patients with potentially more serious diseases should be treated more aggressively than implied by this analysis.
